# Medical Opinion and Movement

**Published:** 1908-08-08

**Authors:** 


### Medical Opinion and Movement.

POLYURIA is often regarded as a nervous symptom, and treated on such a hypothesis. On the other hand, several authors, notably Widal and Ravant, have associated the condition with an excessive consumption of salt and have suggested treatment by dechlorination. Dr. Poisot has recently published an interesting case which bears directly on this question and confirms this view. A healthy, robust man is suddenly affected with intense thirst, and from the time that he was under observation it was ascertained that he excreted from 10 to 12 litres of urine a day. An analysis of the urine showed about 20 grammes of urea and 30 grammes of chlorides in the 24 hours. The phosphate and sulphates were in normal quantities. So long as he took the ordinary diet in the hospital his urinary excretion remained about the same, but when he was subjected to a dechlorinated régime the quantity of urine fell rapidly to about 3½ litres a day. Examination of the urine then showed a daily elimination of only 1 to 2 grammes of chlorides and an amount of urea varying from 20 to 50 grammes. On a return to ordinary diet the polyuria again appeared, to disappear again as soon as the dechlorinated diet was repeated. Moreover, this dechlorination exercised a marked influence on the general condition of the patient. During four months of treatment the patient's weight increased from 65 to 71 kilos. It appears, therefore, that an excessive chlorination produces a definite general disorder of metabolism as well as the polyuria, both of which are, according to the facts of this case, materially influenced by excluding chlorides from the diet.

MUCH time and work have been expended in experimental research on animals with a view to ascertain the chief causal factors at work in man in the production of arterio-sclerosis and atheromatous degeneration. Josué has shown that atheromatous lesions of the aorta can be produced by injections of adrenalin. Alcohol and tobacco are both accredited with the toxic power of inducing arterial degeneration; and several authors have studied the influence of tobacco on the arterial system by submitting animals to injections of tobacco infusions, and of aqueous solutions of tobacco smoke, and by placing them in an atmosphere of tobacco smoke. Gébrovsky has shown that intravenous injections of tobacco smoke in rabbits produce aortic lesions —dilatation of the vessel with thickened patches. More recently, by submitting these animals to an atmosphere of tobacco smoke for several hours each day, they develop anorexia and emaciation, and at the end of a couple of months he finds degenerative changes in the nerve ganglia of the heart and also in the middle and inner tunics of the walls of the aorta. On the other hand, Guillain, who has carried out similar experiments on rabbits, found atheromatous changes in only three out of thirty-three animals so treated. He thinks that certain races of rabbits develop atheromatous changes, either spontaneously or experimentally, much more easily than others, and is of opinion that tobacco is probably an inconsiderable factor in arterial degeneration, and not to be compared with more powerful toxins such as adrenalin. The subject is an important one, and calls for further research on other animals than the rabbit.

ACCORDING to Pers, of Copenhagen, most cases of obstinate sciatica are due to an adhesive perineuritis. He finds that the sciatic nerve in such cases is firmly bound down all along its course by numerous adhesions, chiefly marked around the sacro-sciatic foramen. Bloodless stretching or even forcible "open" stretching is quite insufficient to free the nerve: such methods may break down adhesions in one part and cause a temporary improvement, but the pains invariably return, necessitating further operations. Pers accordingly lays the nerve free along its entire course by an incision which runs longitudinally downwards from the lower edge of the gluteus between the vastus externus and biceps. This incision exposes the nerve, which is then methodically freed from all adhesions, special attention being paid to the foramen, where the nerve is often bound down to the fascia by very dense adhesions. The operation, which is a severe one and not likely to be done in private practice, is not new, and the author gives full credit to Renton, who, in 1898, published a series of cases operated by him, and to Baracz, whose results were published in a Russian medical paper in 1902. In the hands of the Copenhagen surgeon the method has proved eminently successful. Some 47 patients were treated, many of whom had been sufferers for periods varying from ten to forty years. In every case the relief was marked and instantaneous: when the patients awoke from the anæsthetic they felt no longer their old sciatic pain; but only the dull ache of the wound. Permanent results were obtained in 89 per cent. of cases, observed for periods varying from one to five years after the operation. In a few cases the pains recurred and a second operation was necessary. This relieved them of all pain, and Pers ascribes the failure of the primary operation to the fact that the separation was not methodical and thorough enough. The new method is certainly worthy of attention in intractable cases, in which ordinary means fail to give relief.

ALTHOUGH we are probably still a long way off from obtaining a satisfactory cancer cure other than surgical intervention, the efforts which have been made to discover some specific cytolytic substance which would cause a dissolution of the cancer cells have not been altogether fruitless. The work of Sajous, Beard, Von Leyden, and Bergell has demonstrated that certain chemical substances or diastases in the liver and pancreas have a specific influence on cancerous processes. Whether such influence is of the nature of a cyto-inhibition or a pure cancrolysis is not yet clear, but the action of trypsin, for instance, is undoubtedly specific in the sense that neoplastic tissue is less resistant than healthy tissue. Bier, following somewhat different lines, finds that heterologous blood exercises a cytolytic action upon malignant cells, and has used chiefly for this purpose the blood of pigs. These various methods are distinguished as heterodiastasic. On the other hand, it appears that the neoplasms possess also certain diastases, and efforts have been made to activate these diastases against the cells of the neoplasm. Such a method is known as autodiastasic. According to Neuberg the emanations of radium produce their action upon malignant growth in this way. Sticker has endeavoured to induce a similar process by injections of atoxyl. He has succeeded in causing a temporary reduction of lymphosarcomata in dogs, but general intoxication prevents a continuation of the treatment for a sufficient length of time. He has, however, succeeded in causing a complete disappearance of these growths in several dogs by combining injections of atoxyl with Bier's method of blood injections.

---

Dr. Marcel Labbé discusses recently the evil consequences of over-feeding. It may result from habit or choice, or in consequence of medical advice, as in cases of tuberculosis or neurasthenia. The author regards it as one of the commonest causes of ill-health. Obesity, gout, diabetes, and renal lithiasis are some of the later troubles that may result from over-feeding, but it also causes a number of immediate symptoms which make up a characteristic syndrome. The chief of these are gastric symptoms, such as distension and discomfort after meals, sleepiness after meals with insomnia and nightmare at night, an excited mental condition, neurasthenia, and headache. The temperature may be rather above normal, the liver is often hypertrophied, and the urine dark and strong smelling, and may contain some albumin. A systematic comparison between the food ingested and the urine excreted, according to Labbé's method, will demonstrate nutritive disturbances. Over-feeding is further demonstrated by an increased excretion of the normal constituents of the urine by ordinary quantitative analysis. On comparing these results with the food ingested, it will generally be found that there is a deficiency in the absorption of the food by the intestine.

---

Finsterer has devoted a very full and interesting paper to the study of extra-uterine pregnancy, based on a series of 133 cases observed at the Maria Theresia Gynæcological Hospital at Vienna. The work of this young surgeon is always thorough and exact, and we owe to him one of the best of modern contributions to the literature of mammary carcinoma and its operative treatment. It is, therefore, interesting to note his conclusions on this equally important question of extra uterine pregnacy. The first point on which he lays stress is that there is no foundation for the often-repeated statement that such cases are increasing in frequency. The relative increase is due to the fact that better diagnoses are made, and the general practitioner no longer contents himself with the vague definition of "internal hæmorrhage." Finsterer finds that these pregnancies occur most frequently in women between 26 and 30 years of age, the average of all his cases being 27.6 years—a conclusion which is different from that of Martin and Runge, who give a much higher (32 years) average. The youngest patient was 19 years, the oldest 46. The interesting question of ætiology is fully discussed, and the author concludes that in the majority of cases inflammatory conditions of the adnexa are not found, and inclines to favour the view that such pregnancies depend on lesions of the ovum itself. He briefly discusses the side questions, so interesting in themselves, of double extra-uterine pregnancy, due to superfœtation, of which he records some interesting cases, and of primary abdominal gestation. Hitherto gynæcologists have denied the possibility of a primary abdominal pregnancy, but the case recently recorded by Linck in 1900 must be admitted as proving that such pregnancy is possible. Dr. Finsterer's paper abounds in practical hints regarding diagnosis and the operative treatment. Thus to practitioners his summary of the dangers which attend incautious examination of such cases is especially valuable. In several cases, as he shows, palpation under an anæsthetic has led to rupture, necessitating an immediate laparotomy. The method adopted at the Maria Theresia Hospital is to attack the pregnancy by the abdominal route, and extirpate, in early cases, the entire tumour. In more advanced cases the fœtus is removed, the placenta being left to be removed subsequently. In all cases drainage, usually by a gauze wick carried through the pouch of Douglas into the vagina, is employed.

---

At a recent meeting of the medical society of Hamburg Nonne discussed the interesting condition known as "atoxyl blindness." The patient, a woman of 30, received eight grammes of atoxyl during four weeks for inoperable carcinoma uteri. At the end of a month the patient complained of headache and amaurosis. The atoxyl was immediately stopped, but the symptoms rapidly increased and the patient became quite blind. Ophthalmoscopic examination was entirely negative. Two months later the patient died. At the autopsy nothing abnormal was to be seen with the naked eye in the brain or spinal cord. Sections of the optic chiasma and the cuneate nuclei, stained according to the Marchi method, showed evidence of degeneration, but nothing like the interstitial neuritis which Fehr had described. Sections of the cord showed microscopically only a slight degeneration in Goll's column, such as is found in cases of severe intoxications (alcohol, lead, etc.). The case, as the first in which a complete and methodical microscopic examination was made, is interesting but inconclusive, and the findings do not explain the clinical picture. Atoxyl is becoming a popular drug in the treatment of many disorders, and the results of Koch in cases of sleeping sickness have again drawn the attention of the profession to it. It is, therefore, necessary that its dangers should be pointed out. For private practice it is scarcely a good drug, and the question of its chemical purity is one which demands investigation. It is certainly remarkable that in some cases a very small dose of the drug causes toxic symptoms, while in others much larger doses—of a possibly purer sample—may be administered without such symptoms supervening.

THE treatment of the appendix stump at operation is a point upon which opinion varies a good deal on both sides of the Atlantic. Dr. Murat Willis, of Virginia, has collected replies from 105 leading American surgeons as to their practice, and his analysis reveals very much what one expects to find by a similar investigation in this country. Forty-eight both crush and ligature the stump; twenty-nine tie without crushing, thirteen crush but do not tie; seven do one or the other, four do neither. Eleven of the 105 divide the appendix with a cautery, the others use knife or scissors; twenty-eight do not try to disinfect the stump, four use the cautery for this purpose, and the rest use drugs, nearly all carbolic acid. As regards burial of the stump, eleven never do it, eleven usually do it, three have no routine plan, and the rest always bury it, with or without preliminary ligature. Post-operative pain is believed by three to follow burial of the stump, and by two to be greater when it is not buried; the rest notice no difference. There is a very marked impression revealed that serious ill-effects may directly ensue upon non-burial: thus intestinal obstruction, fæcal fistula, peritonitis from leakage, and adhesions are prominent as complications that have been observed to follow the omission of this practice; whereas two surgeons only have noticed such results when the stump has been invaginated. It would be of great interest and value if some surgeon would circularise his colleagues in the United Kingdom over the same question and publish the replies received.

DR. G. B. JOHNSTON, in a paper read before the Johns Hopkins Medical Society, and printed in the *Annals of Surgery*, records his own experience of six cases of splenectomy, together with a collection of all those he can find in surgical literature—including 353 cases considered by Bessel-Hagen in 1900, he is able to report on 708 cases of this operation. He points out very justly that there are two factors which militate seriously against correct deductions from the statistics. One is that successes are much more apt to be published than failures; and the other that hardly anyone gets sufficient practice at a rare operation like this to acquire unusual dexterity or technique. Of so-called idiopathic hypertrophy he concludes that "the indications for removal are not at all absolute"; that is, they depend largely on the size of the spleen and the liability to trauma. The mortality of the operation is just under 20 per cent., and is diminishing. Ectopic spleens seem to be a good deal less dangerous to remove, and their liability to torsion and to adhere to other viscera are held to be good reasons for so dealing with them: when torsion has actually occurred splenectomy is essential, and no death has been recorded in eleven such cases since 1900. The ague-cake malarial spleen is not infrequently removed to prevent rupture or torsion, and the twentieth century mortality of this proceeding seems to be about 13 per cent. In Banti's disease Dr. Johnston finds the mortality of splenectomy to be nearly 20 per cent.; but he is silent as to the effect of successful operation in checking the downward course of the disease. Non-parasitic cysts seem easily and successfully dealt with, for in nineteen cases there was no death. For leukæmia splenectomy is definitely contra-indicated and unjustifiable, a conclusion arrived at some time ago by common consent: probably this operation will not be again attempted. For injuries and wounds immediate operation is declared to be imperative, and it is satisfactory to learn that in 150 reported cases the mortality has been only 34 per cent. Dr. G. G. Ross, be it noted, quoting Berger, whose paper was published in 1902, puts the mortality at 56.7 per cent., and of unoperated cases at 92 per cent. Probably the rate has been diminishing steadily, and it is quite possible that as a result of improved methods of treating shock it may continue still further to improve.

BAER in the *Therap. Monatshefte* discusses diabetic acidosis and the treatment of diabetic coma. In slight cases of diabetes acetone is not eliminated in the urine. The danger which the organism runs in graver cases where there is elimination of acetone, lies partly in loss of nutritive substances, but more especially in the excessive production of acids such as oxybutyric, which are incapable of being burnt up and converted into innocuous substances. The administration of large doses of sodium bicarbonate as soon as this acidosis has manifested itself is not by itself sufficient to overcome coma when this last has set in, although of use in preventing the onset of this condition. Some substance is wanted which, while diminishing acidosis, will overcome also the glycosuria, and it is reasonable to suppose that some such substance will eventually be discovered, for experiments made upon dogs rendered diabetic by the administration of phloridzin, and then treated with glutaric acid have led to results which are very encouraging.

IT was Freund who originally proposed to perform chondrotomy of the first costal cartilage for the purpose of enlarging the superior aperture of the thorax, and rendering the rib mobile, so as to facilitate the respiratory play of the apex of the lung, in cases of early tuberculosis of the apex. The operation is an easy one and free from danger, and can be practised on appropriate cases. Hans Leidel in the *Munch-Med. Woch.* discusses the cases in which the operation is likely to be of benefit. He considers it indicated when there is tuberculous catarrh of the apices, and in the case of adults and young subjects whose superior thoracic apertures are contracted. Should there be ossification and immobility of the first rib without contraction of the aperture, operation would be beneficial. One condition must, however, be fulfilled if the operation is to be successful. The tuberculous process must not have passed beyond the apices of the lungs, for the apices alone can be influenced by the superior thoracic girdle; once the process has advanced to the lower parts of the lung, mechanical conditions of the upper aperture can have no effect. The best results are obtained when the infiltration of the lung-apex is beginning—*i.e.*, when the subclavicular fossa is as yet intact. Hence early diagnosis governs the question of operation. The author points out that the operation is only one of many means to be employed to combat the disease; it merely opens the way to other methods of treatment.

